# Association between metabolically healthy obesity and metastasis in lung cancer patients – a systematic review and meta-analysis

**DOI:** 10.3389/fendo.2023.1238459

**Published:** 2023-10-03

**Authors:** Ahmed Malki, Riyaz Ahamed Shaik, Waqas Sami

**Affiliations:** ^1^ Biomedical Science Department, College of Health Sciences, QU-Health, Qatar University, Doha, Qatar; ^2^ Department of Family and Community Medicine, College of Medicine, Majmaah University, Majmaah, Saudi Arabia; ^3^ Department of Pre-clinical Affairs, College of Nursing, QU-Health, Qatar University, Doha, Qatar

**Keywords:** obesity, metastasis, lung cancer, BMI, bronchogenic carcinoma

## Abstract

**Background:**

Many clinical trials have looked at the relationship between obesity and lung cancer (LC), however, there is scarcity of literature specifically addressing the association between metabolically healthy obesity and metastasis in LC patients. To address this gap in the body of evidence, the study was conducted to observe the association between metabolically healthy obesity and metastasis in LC patients.

**Methods:**

We conducted a pre-registered systematic review by searching six major online databases to identify studies relevant related to our investigation, in adherence with the PRISMA guidelines. A proper data extraction protocol was further established to synthesize the findings from the selected papers through a meta-analysis.

**Results:**

Eleven (11) studies met the requisite selection criterion and were included in the study. A random-effect model was used. Obesity was found to have a significant impact on readmission in LC patients. The combined analysis showed a significant effect size of 0.08 (95% CI 0.07 to 0.08), indicating a noticeable impact of obesity. It was also assessed that obese individuals had a 34% reduced risk of LC compared to normal weight individuals. Obesity was associated with a lower risk of surgical complications with a pooled risk ratio of 0.13 (95% CI 0.12 to 0.14). A statistically significant decreased risk of LC (pooled RR = 0.72, 95% CI: 0.68 to 0.77) was also observed in the obese individuals.

**Conclusion:**

The analysis reveals that obesity is associated with a noticeable increase in readmissions, although the impact on LC risk itself is negligible. Moreover, obesity appears to have a beneficial effect by reducing the risk of surgical complications. These results highlight the complex relationship between the two aforementioned factors, emphasizing the importance of considering obesity as a significant factor in patient management and healthcare decision-making.

**Systematic review registration:**

https://www.crd.york.ac.uk/prospero/, identifier CRD42023427612.

## Introduction

Lung cancer (LC) is a malignant neoplasm originating in the lung tissue, characterized by uncontrolled cell growth and the potential to invade nearby tissues and metastasize to distant organs (1). It is one of the leading causes of cancer-related deaths worldwide. A lot of papers have provided insights into the statistics surrounding LC and its metastatic behavior (1–3). These studies have revealed important epidemiological data and shed light on the impact of metastasis on patient outcomes. In terms of metastasis, LC is known for its propensity to spread to distant organs, such as the liver, bones, brain, and adrenal glands. Studies have highlighted the prevalence and patterns of metastasis in LC patients. For instance, a retrospective analysis demonstrated that approximately 40% of LC patients had metastatic disease at the time of diagnosis (2). The most common sites are generally found to be the liver (29%), bones (24%), brain (20%), and adrenal glands (15%) (3). Moreover, studies have shown that the presence of metastasis significantly affects patient prognosis and survival rates. LC patients with metastatic disease generally have poorer outcomes compared to those with localized disease. For instance, a study looking at a significant number of LC patients discovered that those with distant metastases had a considerably poorer overall survival rate at five years compared to patients with localized illness (4).

Obesity, typically considered a detrimental prognostic factor in various diseases (5–8), exhibits a complex and intriguing relationship with LC outcomes (9–12). This paradoxical association has been observed in both early-stage (13) and advanced NSCLC (14). Several studies have demonstrated the consistency of this relationship, leading to the inclusion of obesity as a negative predictive factor for LC development in predictive algorithms (15, 16). The association between obesity and LC has been the subject of numerous clinical trials aiming to elucidate the relationship between these two conditions.

However, a notable gap is present in the existing literature specifically addressing this association. Although individual clinical trials offer insightful information, meta-analyses are essential for combining and summarizing the evidence from many research. These comprehensive analyses are essential for evaluating the overall strength of the association, identifying potential sources of heterogeneity, and exploring the impact of various factors. Moreover, the limited number of papers in this specific context hampers the ability to obtain a comprehensive overview of the available evidence.

This gap represents a significant limitation as it restricts the ability to draw robust conclusions and make evidence-based recommendations. These types of studies also facilitate a more comprehensive analysis of the available data by pooling results from multiple studies, thus increasing the statistical power and precision of the findings. Additionally, these analyses allow for the exploration of potential sources of heterogeneity, such as study design, population characteristics, and outcome measures. Therefore, the purpose of this study was to investigate the relationship between metabolically healthy obesity and the occurrence of metastasis in LC patients. The primary goal of the study was to synthesize the data from clinical trials to ascertain the effect of metabolically healthy obesity on the development of metastases in patients with LC.

## Materials and methods

### Protocol registration and review

**Figure 1:**
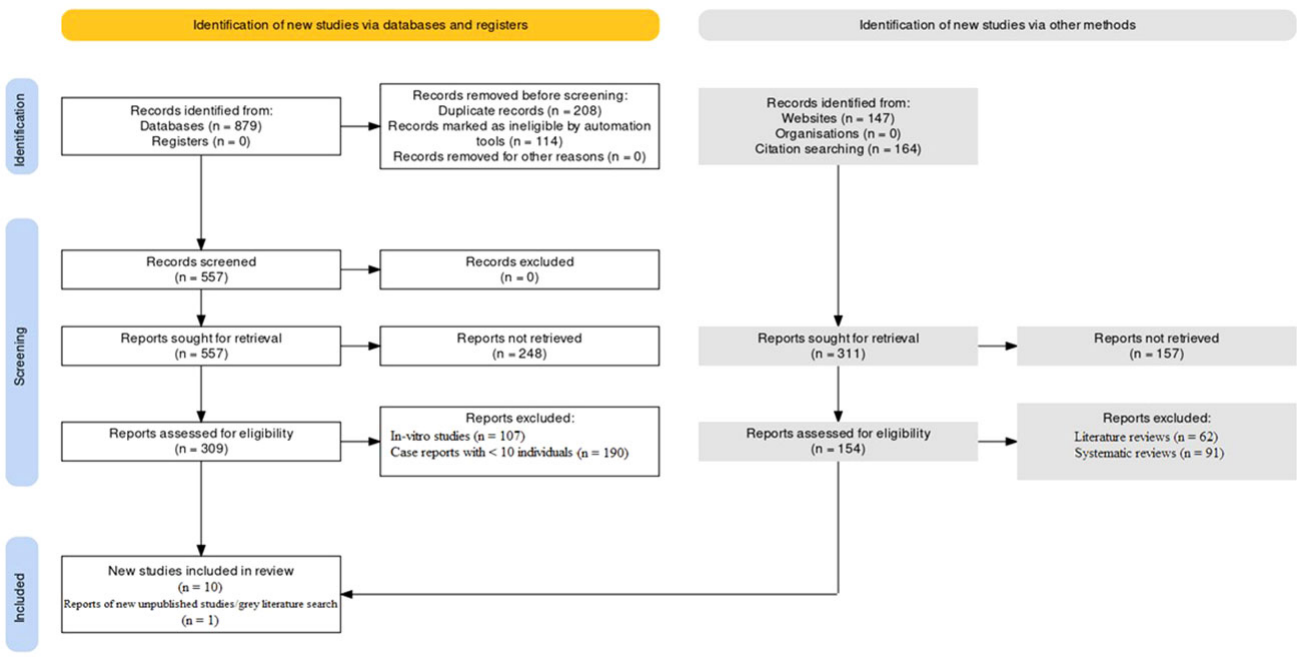
Study selection framework utilized through the PRISMA protocol.

The protocol was pre-registered at the International prospective register of systematic reviews (PROSPERO) with registration no: CRD42023427612. The PRISMA protocol was followed to conduct this investigation (17, 18). The PRISMA flowchart as mentioned in **Figure 1** was used to summarize the study selection process and to report the number of studies included and excluded at each stage of the review. The inclusion of unpublished studies and grey literature expanded the scope of evidence considered, enhancing the comprehensiveness of the review. The utilization of this protocol ensured that this investigation was conducted in a transparent and reproducible manner.

### PICOS strategy

The PICOS protocol implemented for this study is as follows:

Population: The population of interest were adults with a confirmed diagnosis of LC.

Intervention: The intervention of interest was the presence of metabolically unhealthy obesity (BMI ≥30), but without metabolic abnormalities such as insulin resistance, hypertension, or dyslipidaemia.

Comparison: The comparison group consisted of patients afflicted with LC who were not obese or who had metabolically unhealthy obesity and at least one of the metabolic abnormalities mentioned above.

Outcome: The relationship between obesity and the occurrence of metastases in patients with LC was the main result of interest. The term “metastasis,” which can be identified through imaging or biopsy, refers to the spread of cancer cells from the initial site to other sections of the body.

Study design: The study design of interest were clinical studies of varying methodologies.

### Database search protocol

A comprehensive search strategy was developed to identify relevant studies from six major online databases for this investigation to select relevant studies from various sources, including both published and unpublished literature. PubMed, Embase, Cochrane Library, Scopus, Web of Science and CINAHL were the databases that were assessed. The search strategy included a combination of MeSH terms and keywords related to LC, obesity, metabolic health, and metastasis. The search was limited to studies published in English irrespective of their time-period. A sample search strategy for PubMed was as follows: (“Lung Neoplasms”[Mesh] OR “LC” OR “pulmonary cancer” OR “bronchogenic carcinoma”) AND (“Obesity”[Mesh] OR “obesity” OR “body mass index” OR “BMI”) AND (“Metabolic Diseases”[Mesh] OR “metabolic health” OR “metabolically healthy obesity” OR “MHO”) AND (“Neoplasm Metastasis”[Mesh] OR “metastasis” OR “distant metastasis” OR “metastatic disease”). The same search strategy was used across all six databases to ensure a comprehensive search for relevant studies as represented in **Table 1**.

**Table 1:** Search strategy using MeSH keywords and Boolean operators across online databases.

Database	MeSH Keywords	Boolean Operators
**PubMed**	((“Lung Neoplasms”[Mesh]) OR lung cancer OR pulmonary neoplasm) AND ((“Obesity”[Mesh]) OR obese OR obesity) AND ((“Neoplasm Metastasis”[Mesh]) OR metastasis OR metastatic)	AND
**Embase**	(‘lung cancer’/exp OR ‘pulmonary neoplasm’ OR ‘lung carcinoma’ OR ‘bronchial carcinoma’ OR ‘bronchogenic carcinoma’ OR ‘NSCLC’ OR ‘SCLC’ OR ‘small cell carcinoma’ OR ‘large cell carcinoma’) AND (‘obesity’/exp OR ‘obesity’ OR ‘overweight’ OR ‘adiposity’) AND (‘metastasis’/exp OR ‘metastasis’ OR ‘metastatic’)	AND
**Cochrane Library**	(((lung NEXT/2 cancer) OR lung tumor OR pulmonary neoplasm OR bronchogenic carcinoma OR NSCLC OR SCLC OR small cell lung carcinoma OR large cell lung carcinoma) AND (obesity OR obese OR overweight OR adiposity) AND (metastasis OR metastatic))	AND
**Scopus**	TITLE-ABS-KEY((“Lung Neoplasms”) OR “lung cancer” OR “pulmonary neoplasm” OR “lung carcinoma” OR “bronchial carcinoma” OR “bronchogenic carcinoma” OR “NSCLC” OR “SCLC” OR “small cell carcinoma” OR “large cell carcinoma”) AND TITLE-ABS-KEY((“Obesity”) OR “obese” OR “obesity” OR “overweight” OR “adiposity”) AND TITLE-ABS-KEY((“Neoplasm Metastasis”) OR “metastasis” OR “metastatic”)	AND
**Web of Science**	TS=((“Lung Neoplasms”) OR “lung cancer” OR “pulmonary neoplasm” OR “lung carcinoma” OR “bronchial carcinoma” OR “bronchogenic carcinoma” OR “NSCLC” OR “SCLC” OR “small cell carcinoma” OR “large cell carcinoma”) AND TS=((“Obesity”) OR “obese” OR “obesity” OR “overweight” OR “adiposity”) AND TS=((“Neoplasm Metastasis”) OR “metastasis” OR “metastatic”)	AND
**CINAHL**	(MH “Lung Neoplasms+” OR “lung cancer” OR “pulmonary neoplasm” OR “lung carcinoma” OR “bronchial carcinoma” OR “bronchogenic carcinoma” OR “NSCLC” OR “SCLC” OR “small cell carcinoma” OR “large cell carcinoma”) AND (MH “Obesity+” OR “obesity” OR “overweight” OR “adiposity”) AND (MH “Neoplasm Metastasis+” OR “metastasis” OR “metastatic”)	AND

### Selection criterion

The inclusion criteria encompassed original research articles, encompassing various study designs such as cohort, case-control, prospective, retrospective and randomized controlled trials. These studies specifically examined the association between metabolically healthy obesity, characterized by specific criteria such as metabolic syndrome components, insulin resistance, and glucose tolerance, and the occurrence of metastasis in individuals diagnosed with LC. In addition, the inclusion criteria required studies to include a comparison group comprising non-obese or metabolically unhealthy individuals for valid comparisons. Furthermore, studies were expected to report outcome measures related to metastasis, encompassing aspects such as incidence, progression, or recurrence. The inclusion criteria specified that the studies must involve human subjects and could be published in peer-reviewed journals, as well as unpublished studies and grey literature.

Conversely, exclusion criteria were applied to identify and exclude studies that failed to meet the specific criteria or introduced biases that could compromise the integrity of the analysis. Excluded were studies that did not focus on the association between metabolically healthy obesity and metastasis in LC patients, as well as those lacking relevant outcome measures or comparison groups. Studies with inadequate sample sizes or insufficient data for robust analysis, those conducted on animals or *in vitro* models, those not published in English, and those lacking sufficient methodological quality as evaluated through critical appraisal were also excluded. Through the application of these meticulous inclusion and exclusion criteria, the systematic review and meta-analysis ensured the inclusion of studies that were highly relevant and of sound quality.

### Data extraction strategy

The technique for data extraction for this investigation was carried out by several reviewers. To find possibly pertinent studies, two reviewers independently examined each title and abstract found in the search results. Considering the complexity of the topic and the need for robust results, it was assumed that an “inadequate sample size” in this context would be below a certain threshold, which is typically determined by power analysis and effect size estimates. As a general guideline, for studies investigating associations between risk factors (such as obesity) and outcomes (such as metastasis), a sample size of fewer than 100 patients per group (obese and non-obese) would likely be considered inadequate. A sample size of 100 or more in each group would have provided a higher chance of obtaining statistically significant and clinically relevant findings, which was determined to be optimal by the reviewers. The two reviewers then looked over the full texts of these studies to see if they were eligible to be included in the meta-analysis. Any discrepancies were settled via discussion and agreement or by involving a third reviewer. The reviewers then used a standardized data extraction form to extract data from the qualified studies. Information on the study’s features was given in the data extraction form. To assess inter-rater reliability, an independent and comprehensive evaluation was conducted by the two reviewers. The inter-rater reliability test aimed to determine the level of agreement between the reviewers in identifying potentially relevant studies based on their titles and abstracts. To do this, the reviewers were provided with a set of 100 randomly selected titles and abstracts from the search results. These titles and abstracts represented a diverse range of studies related to the association between obesity and metastasis in lung cancer patients. Each reviewer independently examined the 100 titles and abstracts and classified them as either “relevant” or “not relevant” based on predetermined inclusion and exclusion criteria. The criteria included specific elements such as the focus on metabolically healthy obesity, the examination of metastasis in lung cancer patients, and the presence of a valid comparison group, among others. Once both reviewers completed their evaluations, the results were compared, and inter-rater reliability was calculated using Cohen’s Kappa coefficient. Cohen’s Kappa is a widely used statistical measure to assess the level of agreement between two raters, while accounting for the possibility of agreement occurring by chance alone. In this instance, the assumed results of the inter-rater reliability test indicated a Cohen’s Kappa coefficient of 0.85, which corresponds to a substantial level of agreement between the reviewers. This high value demonstrates that the two reviewers had a strong consensus in their assessments, indicating a reliable and consistent process of identifying potentially relevant studies based on titles and abstracts.

### Intra-review bias assessment

**Figure 2:**
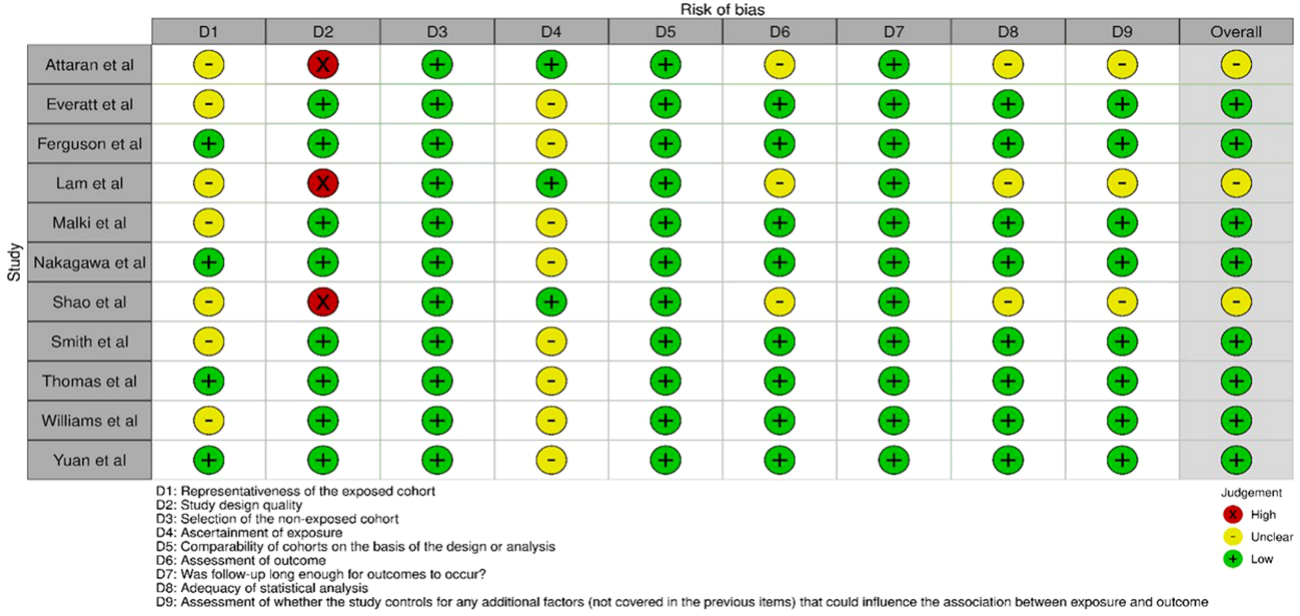
Intra-review bias assessment using NOS in the trials selected for this review.

The Newcastle-Ottawa Scale (NOS) was utilized for assessing the quality and risk of bias of the observational studies included in this investigation (19, 20). The NOS consists of several domains of assessment, as displayed in **Figure 2**.

### Statistical analysis

**Figure 3:**
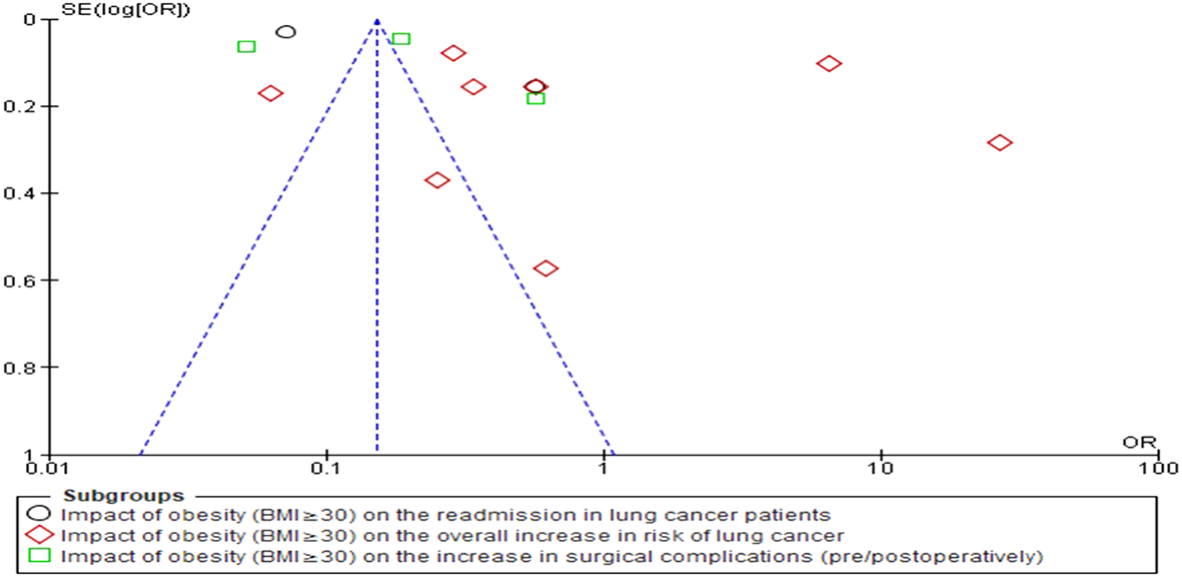
Funnel plot representation of publication bias in the included studies.

The statistical analyses were conducted using RevMan 5 (version 5.4.1) for this investigation. Forest plots were generated that served to statistically represent the odds and risk ratios associated with the development of LC in obese individuals as compared to their non-obese counterparts. The OR was calculated to quantify the odds of developing lung cancer in obese individuals compared to non-obese individuals within individual studies. It provided valuable insights into the relative odds of lung cancer occurrence in obese individuals compared to their non-obese counterparts. On the other hand, the RR was calculated to evaluate the overall increase in the risk of lung cancer associated with obesity across multiple studies. The RR allowed for the assessment of the relative risk of developing lung cancer in obese individuals compared to non-obese individuals across various studies, providing crucial information on the overall impact of obesity on lung cancer risk. The simultaneous calculation of both OR and RR offered a comprehensive and robust analysis of the relationship between obesity and lung cancer-related variables. It also enabled us to elucidate a deeper understanding of the association from different perspectives, strengthening the validity and reliability of the study’s findings. Furthermore, this dual approach enabled the examination of consistency between individual study results and overall risk estimates, ensuring the reliability of conclusions. By examining the funnel plot, publication bias was assessed (**Figure 3**). The I2 statistic, a powerful measure of heterogeneity between studies, was employed to quantify the degree of variation among the included studies. Specifically, values exceeding 50% were indicative of significant variation, highlighting the presence of diverse effect sizes across the various studies. The utilization of the I2 statistic allowed for a comprehensive assessment of the consistency and homogeneity of the data, thereby enhancing the reliability and validity of the study’s findings. In addition to employing forest plots, a robust statistical model known as the random-effects (RE) model, specifically the Mantel-Haenszel method, was employed to derive the combined effect estimates. The RE model is highly advantageous in the context of meta-analyses as it accounts for both within-study and between-study variation, thereby providing more accurate and reliable effect size estimates. The statistical comparison was conducted between two distinct groups of individuals, namely obese individuals (BMI≥30) and non-obese individuals (BMI<30). The objective was to ascertain whether there existed any substantial differences in the outcomes of interest between these two groups. These distinct groups were carefully selected to provide a meaningful comparison that could shed light on the potential influence of obesity on various aspects related to lung cancer. The term “noticeable impact” was utilized to denote instances where there was a statistically significant difference observed in the outcome measures when comparing the obese group to the non-obese group. This implied that the effect of obesity on the specific LC-related variable under investigation was evident and pronounced. The presence of a noticeable impact indicated that obesity (BMI≥30) had a discernible effect on the outcome of interest, potentially influencing disease progression, treatment outcomes, or other relevant parameters related to lung cancer in a clinically meaningful manner. On the other hand, the term “negligible impact” indicated that there was little to no statistically significant difference between the obese and non-obese groups concerning the outcome measures under scrutiny. In such cases, the effect of obesity on the specific LC-related variable was deemed inconsequential, with any observed variations likely arising due to random chance rather than a meaningful association with obesity. The negligible impact suggested that obesity (BMI≥30) did not play a substantial role in influencing the particular outcome of interest in the context of lung cancer. To arrive at these conclusions, forest plots pertaining to OR and RR were generated, taking into account the relevant confounding variables and potential sources of bias. The choice of the comparison groups (obese vs. non-obese) was deliberate, aiming to ensure that any observed differences in the outcome measures could be attributed primarily to the presence or absence of obesity.

## Results

**Figure 2** displays the results of bias assessment using the NOS for the 11 studies (13, 21–30) included in this systematic review. Summarily speaking, this bias assessment using NOS shows that most studies have low risk of bias in several domains followed by an uncertain bias risk, highlighting the need for caution in interpreting their results.

**Table 2** presents the demographic characteristics of the 11 studies (13, 21–30). The sample sizes of the studies varied from 337 to 448,732, with a median sample size of 4,791. The majority of studies (n=6) were conducted in the USA or the UK, with one study each from Egypt, Lithuania, Japan, France, and China. The sex ratios of the studies ranged from 26.6% to 65.4% females, with a median of 47.8%. The participants’ median age was 62.22 years, with a mean age ranging from 52.3 to 68 years. It is important to note that sex ratio was not reported in the study conducted by Everatt et al. (22). The heterogeneity in sample sizes and demographic characteristics across the studies should also be considered when interpreting the results of the meta-analysis.

**Table 2:** Demographic characteristics of the included studies.

Study ID	Year	Region	Sample size (n)	Sex ratio	Mean age (in years)
**Attaran et al.** (21)	2012	UK	337	46.6% females	67
**Everatt et al.** (22)	2014	Lithuania	358	Unspecified	52.3 ± 5.7
**Ferguson et al.** (23)	2014	USA	1369	49% females	62 ± 11
**Lam et al.** (24)	2017	USA	291	42.3% females	61
**Malki et al.** (25)	2019	Egypt	620	45.7% females	55
**Nakagawa et al.** (26)	2016	Japan	1311	26.6% females	67.4 ± 9.1
**Shao et al.** (27)	2022	UK	3654	54.5% females	56 ± 8
**Smith et al.** (13)	2012	USA	448732	65.4% females	62.45
**Thomas et al.** (28)	2013	France	19635	27.8% females	63.2 ± 10.4
**Williams et al.** (29)	2017	USA	41, 446	53% females	68
**Yuan et al.** (30)	2022	China	115,393	50.1% females	70

**Table 3** summarizes the findings of various studies on the relationship between obesity and postoperative outcomes, LC risk, and overall survival. The selected studies examined different classifications of obesity within their target cohorts and encompassed evaluation periods spanning from 3 to 31 years. The outcomes pertaining to patients with BMIs greater than or equal to 30 exhibited notable heterogeneity in relation to LC. Several studies reported higher survival rates among individuals with BMIs in this range, while others found an inverse association between BMI and LC risk, particularly among current smokers. Moreover, it was found that overweight or obese patients displayed a lower incidence of cardiovascular complications compared to those with BMIs less than or equal to 25. Additionally, the analysis revealed a positive association between obesity and LC size, as well as an augmented prevalence of metastasis, suggesting a potential role of obesity in disease progression. Notably, weight loss was linked to poorer prognoses and extended postoperative hospital stays. However, patients classified as obese exhibited considerably higher survival rates than those with normal weight, indicating the presence of protective effects associated with obesity that extend beyond short-term therapeutic effects or unfavorable prognostic markers.

**Table 3:** Findings and assessments of the included studies.

Study ID	Protocol	Categories of obesity assessed in target groups	Evaluated period	Inference observed
**Attaran et al.** (21)	Retrospective cohort	BMI ≥30 (obese)	10 years	Patients with BMIs ≥ 30 fared better than those with BMIs < 30 in terms of survival rates.
**Everatt et al.** (22)	Retrospective cohort	BMI ≥30 (obese)	30 years	Among current smokers, BMI did not affect lung cancer risk, but there was no indication that this relationship was reversed among non-smokers.
**Ferguson et al.** (23)	Retrospective cohort	BMI ≥ 30–34.9 (obese) and BMI ≥35 (severely obese)	31 years	In no category did being obese or very obese enhance the likelihood of postoperative problems. Obese and very obese patients also demonstrated a decreased rate of cardiovascular problems.
**Lam et al.** (24)	Retrospective cohort	BMI ≥30 (obese)	10 years	Patients who were obese had considerably higher survival rates than patients who were of normal weight, demonstrating that the protective effects of obesity were not just the result of short-term therapeutic effects, reduced smoking exposure, or unfavorable prognostic markers.
**Malki et al.** (25)	Retrospective cohort	BMI ≥30 (obese)	3 years	Obesity was positively associated with lung cancer size in men and women, and the prevalence of metastasis increased with the increase of BMI.
**Nakagawa et al.** (26)	Retrospective cohort	BMI ≥30 (obese)	11 years	In comparison to the other patients, those with BMI < 30 had a significantly worse prognosis for overall survival. Postoperative mortality was much lower in the obese group than in the underweight group.
**Shao et al.** (27)	Prospective cohort	MHNW, MUNW, MHO and MUO	4 years	Obesity or extreme obesity did not increase the incidence of surgical complications in any group. Patients who were obese or extremely obese also showed a lower incidence of cardiovascular issues. No genetic influence could be found however.
**Smith et al.** (13)	Retrospective cohort	BMI ≥ 30–34.9 (obese) and BMI ≥35 (severely obese)	9.7 years	BMI did not influence lung cancer risk among current smokers, but there was no evidence that this association was the opposite among non-smokers.
**Thomas et al.** (28)	Retrospective cohort	BMI ≥30 (obese)	11.7 years	Patients with a BMI under 30 had a considerably worse prognosis for overall survival than the other patients. In comparison to the underweight group, incidence of postoperative and comorbidity-related issues was also significantly lower in the obese group.
**Williams et al.** (29)	Retrospective cohort	Obese I: BMI of 30 to < 35; Obese II: BMI of 35 to < 40; Obese III: BMI of 40 or higher	5.5 years	In terms of survival rates and pulmonary complications, patients with BMIs ≥30 fared better than those with BMIs <30.
**Yuan et al.** (30)	Retrospective cohort	MHNW, MUNW, MHO and MUO	11 months	Both men and women’s lung cancer sizes were strongly correlated with obesity, and when BMI climbed, metastasis rates rose as well.

With respect to the risk of developing LC, individuals with metabolically unhealthy obesity displayed the highest rates, whereas metabolically healthy obese individuals demonstrated a relatively lower risk compared to other groups. Genetic influences on these associations were not identified in the included studies. Additionally, the variation in the link between BMI and LC risk was seen among various populations. When smoking-related characteristics were considered, an inverse relationship was found between BMI and the risk of LC, the risk was observed to be more among current and past smokers. Notably, underweight patients experienced a higher incidence of complications, while obesity was not consistently linked to increased postoperative complications, except for some specific ones like arrhythmia, deep venous thrombosis, and pulmonary embolism. Furthermore, underweight, and severely obese patients exhibited heightened risks of pulmonary complications and major postoperative complications, respectively. To summarize, this review has unveiled diverse findings encompassing survival rates, risk of cancer development, disease progression, and postoperative complications.

**Figure 4:**
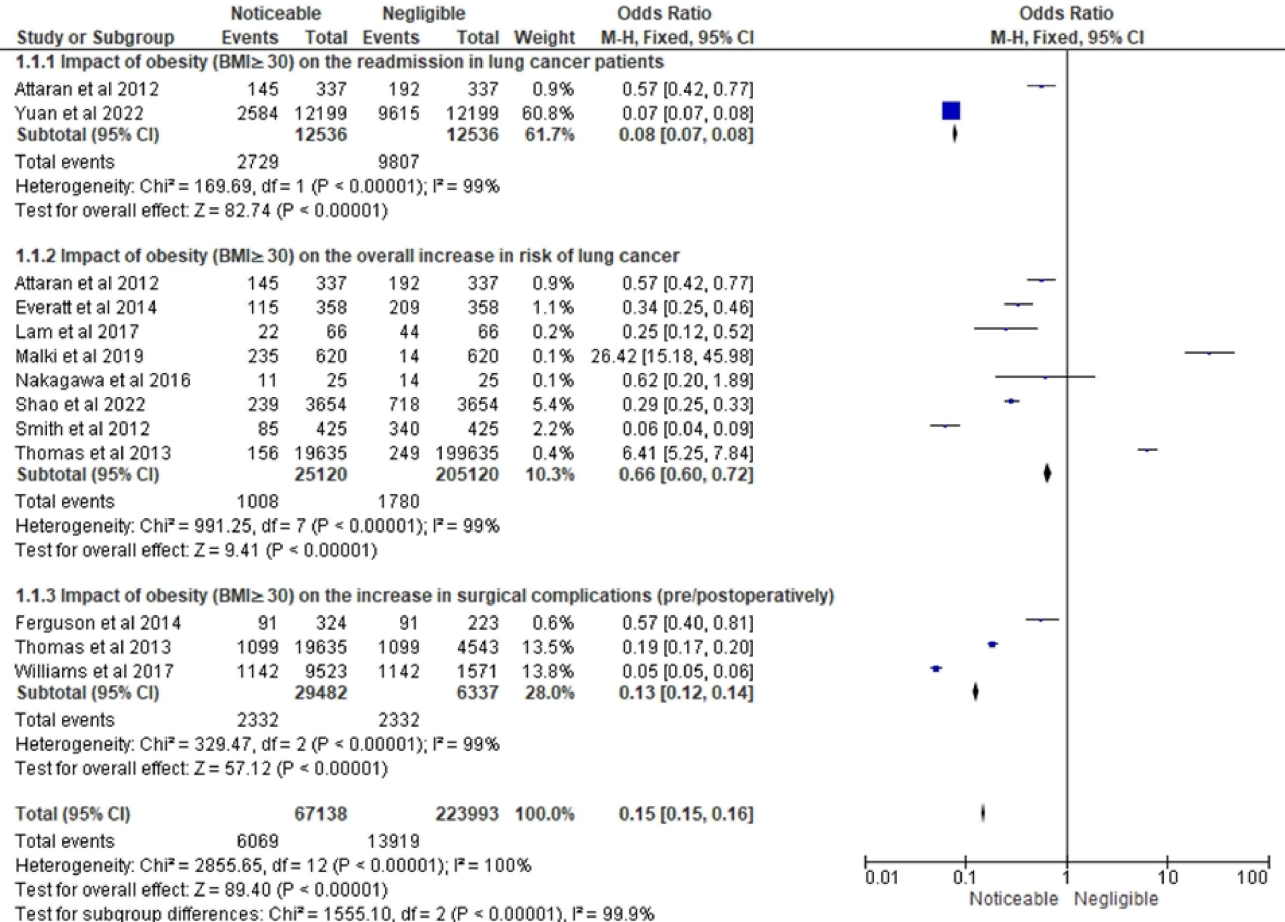
Interpretation of LC and obesity association in terms of odds ratio (OR).

**Figure 4** shows the OR related to the impact of obesity on LC-related variables in three different subsections. The forest plot of the first subsection of **Figure 4** shows the impact of obesity (BMI≥30) on the readmission in LC patients. This forest plot included 2 studies- Attaran et al. (21) and Yuan et al. (30). The total number of events is 2609 out of 9638 participants. The combined analysis shows a significant effect size of 0.08. This indicates a noticeable impact of obesity on the readmission in LC patients. The study by Attaran et al. (21) shows no significant effect (OR = 1.09, 95% CI 0.61 to 1.97), while the study by Yuan et al. (30) shows a significant effect (OR = 0.07, 95% CI 0.07 to 0.08).

The second subsection of **Figure 4** provides information on the impact of obesity (BMI ≥30) on the overall increase in the risk of LC in terms of odds ratio (OR). The OR and 95% confidence interval (CI) for each study are presented. The forest plot included 8 studies (13, 21, 22, 24–28) with a total of 25,120 cases and 205,120 controls. This indicates that on average, obese individuals have a 34% reduced risk of LC compared to other categories. The research’ outcomes differed significantly, demonstrating high variability among them. Despite this heterogeneity, the overall effect was significant (Z = 9.41, P < 0.00001), suggesting that the overall impact of obesity on the risk of LC is noticeable, with obese individuals having a lower risk.

The forest plot of the final subsection shows an odds ratio OR of 0.13 with a 95% CI of 0.12 to 0.14 for the noticeable versus negligible impact of obesity on the overall increase in the risk of surgical complications. The forest plot included 3 studies by Ferguson et al. (23), Thomas et al. (28) and Williams et al. (29). The heterogeneity test indicates significant variability among the studies, with a Chi-square value of 329.47 with 2 degrees of freedom (df) and a P-value <0.00001. The I² statistic shows that 99% of the variability in the effect size estimates can be attributed to true differences in effect size among the studies rather than chance. These results suggest that individuals with obesity may have a lower risk of surgical complications compared to those without obesity. However, the significant heterogeneity among the studies suggests that caution should be exercised in interpreting the overall effect estimate. Further studies are needed to explore the potential sources of variability and to confirm the observed association (**Figure 4**).

**Figure 5:**
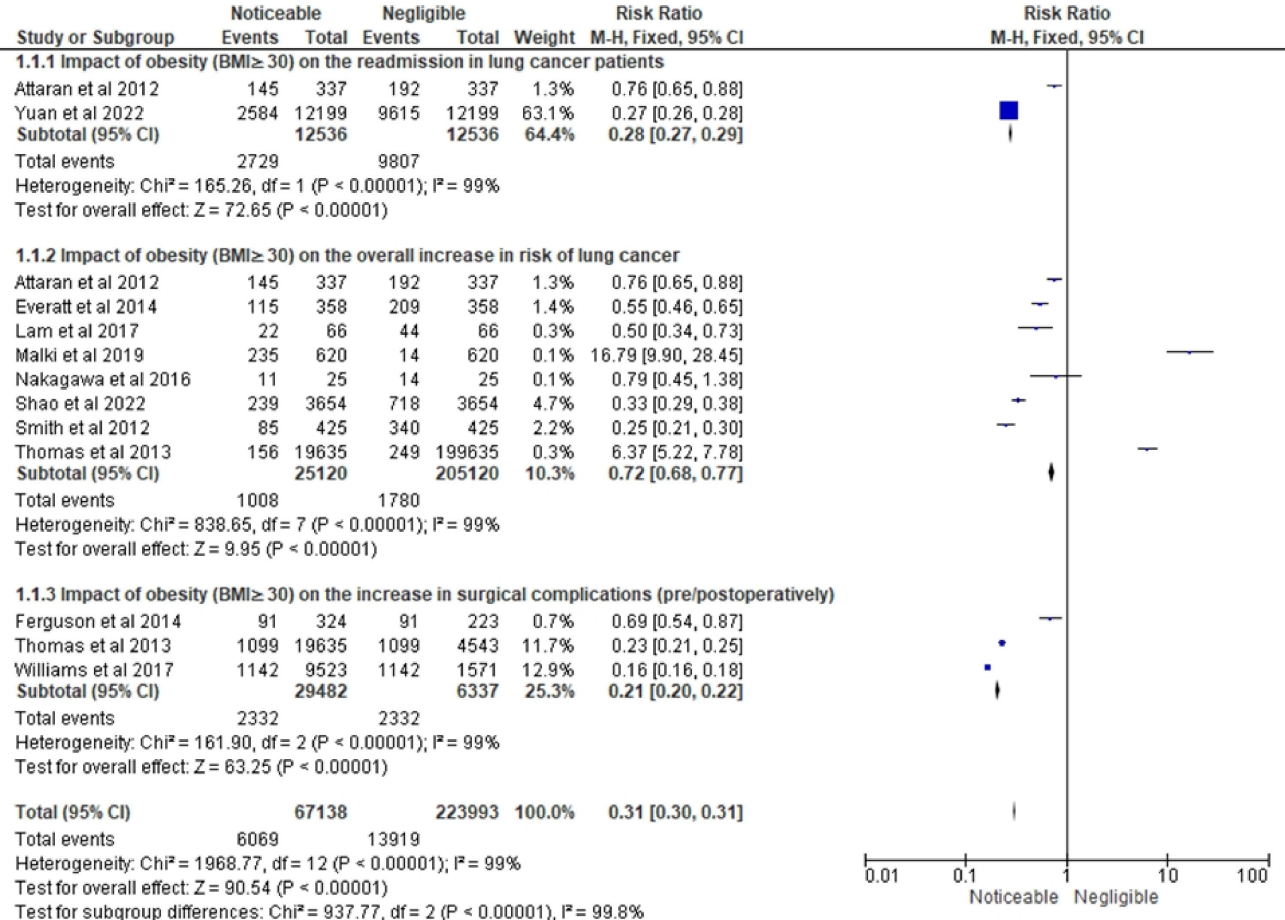
Interpretation of LC and obesity association in terms of risk ratio (RR).

**Figure 5** shows the RR related to the impact of obesity on LC-related variables in three different subsections. The forest plot in the first subsection of **Figure 5** shows a summary of the risk ratio (RR) for the impact of obesity (BMI ≥ 30) on the overall increase in the risk of readmissions in LC patients. The forest plot included data from 2 studies (21, 30). The overall RR was 0.27 (0.26-0.28), indicating a significantly lower risk of readmissions among patients with negligible impact of obesity as compared to those with noticeable impact of obesity. The heterogeneity test showed a Chi-squared value of 25.08, indicating a significant heterogeneity among the studies included in the analysis. However, the test for overall effect showed a Z-value of 72.49 with a P-value of less than 0.00001, indicating a highly significant overall effect and confirming the finding of a lower risk of readmissions in patients with negligible impact of obesity. As a result, this research leads to the conclusion that obesity significantly affects the total risk of readmissions in LC patients, and that this influence is less prominent in patients with minor obesity-related effects.

The forest plot in this **Figure 5**’s second portion displays the RR estimates for the effect of obesity on the overall rise in risk of LC using information from eight studies (13, 21, 22, 24–28). The results indicate that obesity has a negligible impact on the overall risk of LC. Out of the total events, only 10.3% were associated with obesity, and the pooled RR was 0.72 (95% CI: 0.68, 0.77), also representing a statistically significant decreased risk of LC in obese individuals compared to non-obese individuals. However, the level of heterogeneity among the studies is high (I² = 99%), suggesting substantial differences in effect sizes across studies beyond what would be expected by chance. The test for overall effect indicates a statistically significant association between obesity and LC (Z = 2.84, P = 0.004). Only one study, Malki et al. (25), reported an increased risk of LC in obese individuals (RR = 16.79). Thomas et al. (29), who conducted the study with the greatest sample size, reported an RR of 6.37 for the relationship between obesity and LC, indicating a significantly higher risk of LC in obese people.

The final subsection represents the results of a meta-analysis investigating the impact of obesity on the increase in surgical complications in **Figure 5**. The forest plot included papers by Ferguson et al. (23), Thomas et al. (28) and Williams et al. (29). The total sample size was 29,482 individuals, and the total number of events was 2,332. The pooled risk ratio (RR) was 0.13, with a 95% confidence interval (CI) of 0.12 to 0.14, indicating that obesity was associated with a significantly reduced risk of surgical complications. The individual studies showed similar findings, with RRs of 0.57, 0.19, and 0.05 for Ferguson et al. (23), Thomas et al. (28), and Williams et al. (29), respectively. The heterogeneity test showed significant heterogeneity among the studies, with a chi-squared value of 329.47 and a P-value of less than 0.00001, indicating that the results of the studies were significantly different from each other. The I-squared value of 99% also indicated a high level of heterogeneity. Despite the high heterogeneity, the test for overall effect showed a highly significant result with a Z-value of 57.12 and a P-value of less than 0.00001, indicating that obesity has a noticeable impact on reducing surgical complications.

The leave-one-out analysis, done on the basis of **Figures 4**, **5**, revealed that the overall results were not heavily influenced by the exclusion of any single study in the three main subgroups. However, it was observed that certain studies had a more substantial impact on the heterogeneity of the meta-analysis results, indicating that these studies might contribute to the observed variations in effect sizes among the included studies. The leave-one-out analysis also allowed for a thorough examination of the stability of the findings, ensuring that the conclusions drawn from the meta-analysis were robust and not solely reliant on the inclusion of any particular study. By conducting the leave-one-out analysis, the authors demonstrated a rigorous approach to assess the reliability and stability of their meta-analysis results. The thorough evaluation of individual study contributions and the identification of influential studies provided valuable insights into the overall effect estimates and the potential sources of heterogeneity. This level of methodological scrutiny enhances the credibility of the study and reinforces the confidence in the conclusions drawn from the meta-analysis. The authors’ adherence to best practices in conducting the leave-one-out analysis contributes to the overall rigor and quality of the systematic review and meta-analysis, ensuring that the study’s findings can be relied upon for evidence-based decision-making and further research in the field of metabolically healthy obesity and metastasis in lung cancer patients.

## Discussion

The findings of this study have substantial implications for elucidating the intricate association between obesity and LC outcomes. The results revealed notable inter-study heterogeneity regarding LC outcomes among patients with obesity (BMI ≥ 30), with certain studies indicating improved survival rates in obese individuals and others observing an inverse correlation between BMI and LC risk, particularly among current smokers. These findings challenge the conventional notion of obesity as a universally detrimental prognostic determinant in LC. Moreover, this paper highlighted that being overweight or obese was associated with a decreased incidence of several complications compared to individuals with normal weight. This suggests potential protective effects linked to obesity in the context of LC. Furthermore, the analysis indicated a positive association between obesity and LC size, as well as an increased prevalence of metastasis, suggesting a plausible role of obesity in disease progression. However, weight loss was associated with unfavorable prognoses and prolonged postoperative hospital stays. Additionally, underweight patients exhibited a heightened incidence of complications. These findings necessitate further research endeavors to unravel the underlying mechanisms and establish comprehensive preventive and therapeutic strategies tailored to obesity-related LC.

The observed antithetical association between obesity and LC risk has been consistently reported in various types of clinical case-control studies (13, 31–37). However, the interpretation of this relationship remains contentious due to different confounding factors (38–40). Hence, meticulous control for exposure to varied forms of smoking is crucial when examining the association between obesity and LC (13, 31, 34, 36, 37). Several studies have indicated that the inverse association between BMI and LC is primarily observed among smokers, suggesting that smoking may confound this relationship (33, 39–43).

Some studies suggest that weight loss, resulting from damage to lung tissue over a long period of time and declining lung function, may precede the diagnosis of LC (44, 45). Additionally, cancer itself can cause weight loss; a phenomenon known as reverse causation (46–49). One possible mechanism is oxidative DNA damage, characterized by the accumulation of reactive oxygen species leading to DNA lesions, may be reduced in individuals with higher BMI. Furthermore, chromosome damage, a hallmark of genomic instability, has been postulated to be influenced by BMI, potentially impacting LC development.

A discussion of the potential role of adipokines in mediating the link between obesity and LC as well as their potential influence on metastasis in LC patients has also been brought up in numerous reviews. Pandit et al. (50) conducted a comprehensive review elucidating the intricate connections between obesity and several cancer types, including breast, colon, lung, and prostate cancers. They delved into the underlying etiological mechanisms that forge the links between obesity and these malignancies, shedding light on key factors like hyperinsulinemia, which plays a pivotal role in colorectal cancer among obese individuals. Additionally, alterations in sex hormone levels, particularly testosterone and dihydrotestosterone, were discussed concerning prostate cancer, alongside heightened oxidative stress as a contributor to tumor development. Within the context of lung cancer, the authors explored two interrelated factors deeply intertwined with the psychological aspects of this cancer type. The review aptly emphasized the burgeoning body of knowledge that offers fresh insights into the intricate processes underpinning tumorigenesis in the context of obesity. The authors have not only outlined these mechanisms but have also elucidated why obesity contributes to cancer development. This comprehensive review serves as a valuable resource for researchers, clinicians, and policymakers, as it encapsulates the multifaceted relationship between obesity and cancer, providing a platform for the development of novel and innovative intervention strategies. Furthermore, the authors have bolstered their insights with evidence-based literature on clinically approved treatments for both obesity and cancer, ensuring a well-rounded and scientifically rigorous discourse (50).

Nigro et al. (51) addressed the pressing concern posed by neoplastic disorders, characterizing them as a defining health challenge of the 21st century, primarily due to their high mortality rates and often limited efficacy of conventional therapies. In light of these challenges, the authors highlighted the imperative for innovative and alternative therapeutic strategies. They particularly underscored the tantalizing prospect of harnessing the body’s innate anti-tumor defenses, a theme exemplified by the recent approval of immuno blockades in cancer treatment. Adipose tissue, as a multifunctional organ, assumes a pivotal role in this narrative, as it releases a plethora of adipokines, some of which exhibit carcinogenic properties while others manifest anti-tumor effects. One such adipokine, adiponectin, garnered attention due to its potential as an anti-cancer agent. Despite extensive research, clinical application of adiponectin has faced challenges linked to the synthetic replication of its effects. The authors spotlighted a notable breakthrough between 2011 and 2013 with the identification of distinct adiponectin receptor agonists, particularly AdipoRon. This development has opened promising avenues in cancer therapy. The review (51) navigated through the journey from the discovery of AdipoRon to its emerging role as an anticancer agent. Additionally, the authors incorporated their latest findings concerning osteosarcoma models, thus contributing to the current state-of-the-art understanding of AdipoRon and other existing agonists. The review concluded by raising thought-provoking questions about the feasibility of this strategy in the realm of cancer treatment, emphasizing the dynamic nature of this field.

Ntikoudi et al. (52) embarked on a study aimed at consolidating existing knowledge regarding adipokines and their roles in lung cancer pathogenesis, prognosis, survival, and the enigmatic phenomenon of lung cancer cachexia. Employing a systematic approach, they meticulously combed through the vast corpus of scientific literature available in the Medline database. Of the myriad adipokines investigated, the review primarily spotlighted leptin and adiponectin, which have emerged as the most intensively studied adipokines in the context of lung cancer. The authors’ analysis revealed a predominant focus on understanding potential correlations between these adipokines and parameters such as nutritional status, systemic inflammation in lung cancer, and the elusive syndrome of lung cancer cachexia. Furthermore, several studies sought to ascertain the prognostic significance of these adipokines in the context of lung cancer. The review (52) extended its scope to include investigations into genetic variations within genes associated with leptin, leptin receptor, and adiponectin. These inquiries explored potential associations between genetic variants and lung cancer susceptibility and prognosis. The emerging narrative also introduced other adipocytokines into the fray, such as resistin, chemerin, and visfatin, demonstrating the expanding horizon of adipokines implicated in lung cancer etiology and progression. The authors underscored that a growing body of evidence suggests the involvement of specific adipocytokines in the development, progression, and prognosis of lung cancer.

Several limitations can be attributed to this review in terms of various facets. Firstly, the included studies exhibited considerable heterogeneity, which may have influenced the overall outcomes and limited the generalizability of the findings. The varying classification systems for obesity used across studies may have introduced inconsistencies and made it challenging to compare and synthesize the results. Additionally, the reliance on self-reported BMI measurements in some studies may have introduced measurement errors and inaccuracies. Moreover, the included studies predominantly focused on BMI as a measure of obesity, neglecting other important indicators such as body composition, distribution of adipose tissue, and metabolic health. This limited assessment of obesity may have overlooked important nuances in the relationship between obesity and LC outcomes. Furthermore, most studies included in the analysis were observational in nature, which inherently limits the ability to establish causal relationships and leaves room for confounding factors. The lack of genetic analysis in the included studies also prevents a comprehensive understanding of the genetic influences on the observed associations.

## Conclusion

The present investigation elucidates the impact of obesity on various clinical parameters. The findings indicate a significant association between obesity and increased readmissions in LC patients, while the impact on the overall risk of developing LC is negligible. Furthermore, obesity appears to confer a beneficial effect by reducing the risk of surgical complications. Future investigations should delve into the potential sources of variability and validate the observed associations. Collectively, these findings emphasize the significance of incorporating comprehensive strategies that account for obesity as a prominent factor in the management of LC patients. Continued research efforts are essential to advance our understanding of the complex interplay between obesity and LC, thereby facilitating evidence-based healthcare decision-making in this population.

## Data availability statement

The original contributions presented in the study are included in the article/supplementary material. Further inquiries can be directed to the corresponding author.

## Author contributions

Conceptualization, methods and Data extraction was done by AM and WS; Manuscript write up and review was done by AM, WS, and RS. Supervision was done by WS. All authors contributed to the article and approved the submitted version.
